# Notes and News

**Published:** 1904-10-01

**Authors:** 


### NOTES AND NEWS.

Hong Kong is now reported free of plague.

Complete fire protection arrangements have been introduced at the Royal Portsmouth Hospital.

Sir James Reckitt has offered to double the amount of any Hospital Sunday Fund collection made in Hull after the first thousand pounds has been obtained.

The last report from Dewsbury showed that the number of small-pox cases were increasing, but no news of an official character is at present forthcoming, as reports to the Press have been discontinued. Oldham and Ashton have also experienced epidemics of a smaller character since August.

The Liverpool School of Tropical Medicine is about to despatch a second yellow-fever expedition to the Amazon. In the first expedition both medical commissioners were attacked with the malady.

The new Branch Hospital in connection with the Mount Vernon Hospital for Consumption is now open at Northwood. The institution is the gift of an anonymous donor, and has cost about £100,000. The site is a fine one, and all the wards face south.

Dr. Leith Napier, whose connection with the Adelaide Hospital will be remembered, has made a claim against the South Australian Government of £6,500 for injuries received in military service. He volunteered for South Africa, and sustained severe injury from a fall from his horse before departure.

Three Poor law infirmaries have just been added to the pauper accommodation of Glasgow. The largest of these infirmaries is the General Hospital at Stobhill for 1,650 patients, and where consumptives will be received. The Eastern District Hospital is in Duke Street, Glasgow, and will receive mental cases amongst its 244 patients. The Western District Hospital is at Garscube Toll, and provides for 207 patients. The plans, with a full description of the Eastern District Hospital, were published in our columns on June 20th, 1903.

The Consumption Sanatorium at Stanhope in the county of Durham has been extended, and will now accommodate 44 patients in place of 20. The cost of the extension has been £1,200.

Saturday, October 15th, is the date fixed for the annual "special" collection of the Hospital Saturday Fund, which takes the place of the former street collection. The weekly system of subscriptions will, of course, be continued until the end of December.

A bazaar is to be held in October at Battersea on behalf of the Bolingbroke Hospital, Wandsworth Common. Hospital accommodation for South Londoners in this locality is much needed, and it is hoped to so enlarge the Bolingbroke Hospital as to meet the want. £30,000 will be required.

Mrs. Humphry Ward and others have done much to improve the lot of defective children by the establishment of special schools. Berlin has united to much the same scheme a species of outdoor treatment during the summer months. The institution is known as the Forest School, and here amongst the pine trees, education is carried on in an open shed, meals are provided, and rest and recreation are arranged for. The actual time for study does not exceed more than two and a-half hours daily. A medical man visits the school regularly.

We are glad to see that *Truth* is on the track of the "Penny Fund" for the hospitals which is being advertised in *Public Opinion*. It has blossomed out in the columns of that paper as a new scheme, but in reality it is the same objectionable enterprise which was exposed by *Truth* in the spring, and upon which we commented in our columns on May 14th. A scheme to raise money on a commission basis of 25 per cent. must be regarded as a good financial arrangement for the promoters rather than a commendable one either for the public or the charities which it is proposed to benefit.

Macclesfield Infirmary, which suffered recently from an absconding secretary, has appointed a new officer. The institution promises to be open to congratulation. Mr. Hanrahan, who is to combine the offices of secretary and dispenser, has been quartermaster-sergeant in the Royal Army Medical Corps for 21 years, and is experienced in the conduct of hospital business and dispensing. He is pensioned, and for this reason we conclude the committee has been able to secure so experienced an officer to accept such arduous work for the small salary of £100 per annum. This sum, by the way, is an increase on the salary received by the gentleman who disappeared. We think that a good many institutions have yet to learn that the skilled labourer should receive fair remuneration, whether he is working in the cause of charity or not. The combination of reliable dispensing and good book-keeping, together with the other numerous items of a secretary's duties, are worth more than £100 per annum, and it is improbable that a skilled and trustworthy candidate for the post could be obtained unless he already possessed an income from other sources.

